# Household Costs of Leprosy Reactions (ENL) in Rural India

**DOI:** 10.1371/journal.pntd.0003431

**Published:** 2015-01-15

**Authors:** David J. Chandler, Kristian S. Hansen, Bhabananda Mahato, Joydeepa Darlong, Annamma John, Diana N. J. Lockwood

**Affiliations:** 1 Department of Clinical Research, London School of Hygiene and Tropical Medicine, London, United Kingdom; 2 Department of Global Health and Development, London School of Hygiene and Tropical Medicine, London, United Kingdom; 3 The Leprosy Mission Home and Hospital, Purulia, West Bengal, India; 4 The Leprosy Mission Trust India, New Delhi, India; Emory University, UNITED STATES

## Abstract

**Background:**

Erythema nodosum leprosum (ENL) is a common immune-mediated complication of lepromatous (LL) and borderline lepromatous (BL) leprosy. Most patients experience chronic or multiple acute ENL over many years during an economically active period of their lives. Understanding the economic burden of ENL is essential to provide effective patient support, yet this area has not been investigated.

**Methods:**

Ninety-one patients with LL or BL leprosy attending a leprosy hospital in Purulia district of West Bengal, India, were interviewed using a structured questionnaire. Cases (n = 53) were identified as those who had one or more episodes of ENL within the last 3 years. Controls (n = 38) had LL or BL leprosy but no history of ENL. Data were collected on household income, direct and indirect costs, and coping strategies.

**Findings:**

The total household cost was Rs 1543 per month or 27.9% (IQR 13.2-52.6) of monthly household income for cases, and Rs 237 per month or 4.9% (IQR 1.7-13.4) of monthly household income for controls. Indirect costs accounted for 65% of total household costs for cases. Direct costs accounted for the remaining 35% of household costs, and resulted almost entirely from treatment-seeking in the private sector. Total household costs exceeded 40% of household income for 37.7% of cases (n = 20) and 2.6% of controls (n = 1) [1 USD = 59 INR].

**Interpretation:**

Households affected by ENL face significant economic burden and are at risk of being pushed further into poverty. Health policy should acknowledge the importance of private sector provision and the significant contribution to total household costs of lost productivity (indirect cost). Further work is needed to explore this area and identify solutions.

## Author Summary

Erythema nodosum leprosum (ENL) is a common complication of leprosy and an important cause of nerve damage and disability. In most cases, ENL causes chronic or recurrent episodes of ill-health over many years. In this study, we show that having a family member affected by ENL places considerable financial burden on households in rural India. Household costs resulted predominantly from the impact of ENL on the productivity (ability to earn money) of household members. Out of pocket expenditure on treatment-seeking in the private sector accounted for the remaining costs. Leprosy affects poor and marginalised communities in low- and middle-income countries across the world; households affected by ENL are at risk of being pushed further into poverty. The findings of this study support the need to better understand ENL and develop improved strategies for the prevention and management of ENL.

## Introduction

Leprosy is a disease of poverty, affecting poor and marginalised communities in tropical countries throughout the world.[1] More than 200,000 new cases of leprosy are registered worldwide annually, with 60% in India. Leprosy reactions occur in up to 50% of patients with multibacillary leprosy and cause nerve damage and disability.[2,3] Two types of reaction can occur: type 1 reaction (T1R) and type 2 reaction (T2R) or erythema nodosum leprosum (ENL). At the end of the first quarter of 2014 there were 180,618 leprosy patients on record for treatment globally (estimated prevalence rate 0.32 per 10,000 population)[4] however this does not include those patients who have completed treatment and are at risk of developing reactions.

The lepromatous (LL) and borderline lepromatous (BL) forms of leprosy are characterised by low cell-mediated immunity to the causative organism *Mycobacterium leprae*. There is uncontrolled proliferation and dissemination of leprosy bacilli resulting in extensive infiltration of the skin and other organs. Erythema nodosum leprosum (ENL) is an immune-mediated complication affecting patients with LL and BL leprosy. Patients with ENL have widespread crops of tender, erythematous swellings in the skin, and other organs are often inflamed. The physical impact of ENL is significant—patients suffer repeated episodes of ill health over many years and many develop impaired organ function, deformity and disability.[5] Patients need prolonged courses of high-dose steroids or thalidomide to control inflammation, and this can cause adverse effects including steroid dependency.[6]

Families with ENL are affected by out of pocket expenditure for treatment-seeking (direct cost) and loss of income resulting from reduced productivity (earning potential) of household members (indirect cost). There are some data on the economic burden of malaria and visceral leishmaniasis on households in low-income countries,[7–11] however none for leprosy. Understanding the economic burden of ENL is essential to provide effective patient support and to inform researchers, healthcare professionals, health policy makers and managers of control programmes.

Qualitative research has provided some insight into the social and economic consequences of leprosy; however quantitative data on the economic impact of leprosy and ENL do not exist. The Social Welfare Department of the Government of Delhi, India, operate financial assistance schemes for the families of patients affected by leprosy. This financial support is helpful for eligible families, however the schemes are based on a limited understanding of the costs affecting households, have no impact on preventing or reducing these costs, are neither suitable nor efficient long-term solutions, and may not necessarily be available to the families who are most in need of support (many do not have bank accounts). Socio-economic rehabilitation (SER) and microfinance programs can provide much needed financial support for leprosy patients and their families.[12,13] The International Federation of Anti-Leprosy Associations (ILEP) has produced guidelines which identify the broad principles of effective SER programmes, however the design of such programmes would greatly benefit from detailed quantitative data on household costs. Understanding the specific problems faced by families affected by ENL and how costs arise will allow the development of solutions that are appropriate to the needs of the target population. Understanding the magnitude of economic burden on households will predict the degree of financial support required by families and ensure that interventions for ENL are socially viable (any costs to the patient are financially acceptable).

In this study we determined the economic impact of having a family member with erythema nodosum leprosum (ENL) on households in rural India. We estimated direct and indirect costs associated with ENL and expressed these in relation to household income, and investigated the coping strategies used by households. We hypothesised that patients with lepromatous (LL) and borderline lepromatous (BL) leprosy complicated by ENL would incur greater household costs than those without ENL.

## Methods

### Ethical considerations

Ethics approval was obtained from the London School of Hygiene and Tropical Medicine (LSHTM) MSc Research Ethics Committee (ref 012–275) and from the ethics committee of The Leprosy Mission (TLM) Trust India, for this study to be carried out in TLM hospital Purulia, West Bengal, India. Informed written consent was provided by all adult patients or the parent or guardian if the patient was a child.

### Study design

The perspective of this study was microeconomic (households). We aimed to determine the economic impact of ENL on families. We assessed losses associated with the market economy only, providing estimates of direct costs and indirect costs (lost market production). These costs were combined to provide an estimate of the net impact of ENL on households’ opportunities to consume non-health goods and services (the primary outcome of interest). An appropriate counterfactual (control patients with LL/BL leprosy but no history of ENL) was used for comparison of cost data. The broader effects of ill-health on economic welfare, including reductions in health status and leisure time, were not considered in this analysis. The cost to the rest of the society of ENL was not explored.

### Patient selection

All patients with a diagnosis of lepromatous (LL) or borderline lepromatous (BL) leprosy were eligible for inclusion in the study. Cases had LL or BL leprosy and one or more episodes of ENL within the last 3 years. Controls had LL or BL leprosy but no history of ENL. Consecutive patients attending the hospital outpatient department and those admitted to the hospital during the study period (19^th^ June—27^th^ July 2013) were recruited. Published case definitions for ENL were used.[5] Erythema nodosum leprosum (ENL) was defined as an eruption of tender erythematous skin nodules; fever and evidence of other organ inflammation may be present. Single acute ENL was one episode lasting less than six months with no recurrence of ENL while receiving prednisolone, no increase in severity requiring an increased steroid dose and no recurrence after the prednisolone has stopped. Multiple acute ENL was more than one episode with the same characteristics as acute single ENL. Chronic ENL was an episode lasting more than six months and could include single and multiple episodes.

### Data collection

Data were obtained using a structured questionnaire, administered to the patient or to the parent or guardian if the patient was a child. All interviews were conducted in a private and informal setting in the hospital outpatient department, by the first author (DC). Translation services were provided by an existing member of staff at TLM Purulia (BM). Patients were approached in the hospital waiting room, in between appointments with different members of hospital staff, and were recruited and interviewed with minimal delay. The objectives of the study were outlined to patients before starting the questionnaire, and it was explained that there would be no financial reward or adjustment to treatment costs based on the responses provided during the interview. Patients were generally enthusiastic to participate and help with the study. The questionnaire was piloted on 8 eligible patients (5 cases, 3 controls) allowing improvements to be made to questionnaire content. The results from the pilot interviews were not included in the analysis. Patients were interviewed once only. The questionnaire collected data on clinical history, household income and socioeconomic status, treatment-seeking behaviour, direct and indirect costs, and coping strategies. Direct and indirect costs were specific to leprosy; the presence of other comorbidities and their associated costs was not ascertained. Data were gathered on the number of episodes and type (chronicity) of ENL.[5] The clinical history provided the duration of ill-health (time since diagnosis of leprosy for controls, time since first episode of ENL for cases) and in doing so defined the time dimension or recall window for which cost and income data were captured. These data were collected retrospectively for the full duration of ill-health, up to a maximum of 3 years. Total costs for the whole recall window were converted to an average cost per month and presented as a proportion of monthly household income. This allowed costs to be compared between patients with varying duration of ill-health.

The occupations of all economically active household members and their daily cash earnings were reported by the patient, including regular cash income from family members not living at home. We defined the household as all persons living in the housing unit and those persons not living at home but who contribute to the generation and/or consumption of household wealth. All agricultural output was considered to contribute to income regardless of whether the products were sold or consumed within the household. All government income support (pension and disability allowance) was included in estimates. Socioeconomic status was assessed using place of residence (urban or rural) and household asset data. The household assets were the same as those recorded in the National Family Health Survey (NFHS-3) India, 2005–2006.[14]

### Direct costs

Data were gathered on treatment-seeking behaviour during the period of ill-health. Medical and non-medical costs were recorded per visit, for all healthcare providers visited. Medical direct costs included provider fees (for consultations and hospital admissions) and payment for investigations, medicines and other treatments. Multidrug therapy (MDT) was available to patients free of charge from all health providers, however not all providers received a free supply of MDT. The cost to these providers of procuring MDT was not determined. Non-medical direct costs included transport costs to and from the health facilities and any additional expenditure on food and other non-medical goods or services. Invoices were available electronically for all inpatient and outpatient episodes at TLM Purulia, and provided an exact breakdown of the medical costs (as listed above) for each episode. Invoices also included the total medical cost per episode (unadjusted cost) and the amount the patient could afford to pay (adjusted cost). The difference between these two values was the subsidy provided (cost incurred) by the hospital for that episode. Cost data presented in this paper are adjusted unless otherwise specified, as these represent the actual costs incurred by the family (the outcome of interest). Unadjusted costs are included for completeness and these represent the amount the family should have paid if they were able to (without subsidised treatment costs at TLM Purulia). We defined catastrophic health expenditure as adjusted direct costs in excess of 40% of total household income.

### Indirect cost

Indirect costs refer to the reduction in household productivity resulting from the interruption of normal or preferred activities of household members. We used an output-based approach to value the time diverted as a result of ill-health in all persons contributing to the economic productivity or wealth of the household. For those with formally-paid wages, the indirect cost was the daily earnings multiplied by the number of days lost. The contribution of individual household members to agricultural output was not determined. Study participants were asked to report the overall loss of income from agriculture during the period of ill-health. When provided with information on the reduction in agricultural output local market prices were used to determine the monetary value. The market values reported by patients were remarkably consistent. The use of an output-based approach was appropriate in this setting, allowing us to account for the significant fluctuations in work intensity that occurred during the year and the coping strategies used to minimise productivity losses, for example recruitment of additional labour to minimise farming losses. The cost of hiring external labour was valued using average local wage rates for males and females. Coping strategies used to generate money, for example selling assets or taking a loan, were identified but considered separately and not factored into cost data. Time diverted by those engaged in unpaid production (children, the elderly, jobseekers and women engaged in domestic work) was considered valueless for the purpose of this analysis and excluded from estimates of indirect costs.

### Statistical methods

Data were analysed using Minitab 17. Household income and cost data are reported using medians and interquartile ranges. The Mann-Whitney (Wilcoxon ranksum) test was used to compare differences in costs between cases and controls. 1 USD = 59 INR; Bloomberg 19 June 2013.

## Results

### Clinical details

Ninety-one patients (53 cases and 38 controls) were identified and all agreed to be interviewed for the study. There were no drop outs at any stage. All patients had lepromatous (n = 53) or borderline lepromatous (n = 38) leprosy. Clinical details are shown in table 1.

**Table 1:** Patient characteristics and clinical details.

		**Cases (n = 53)**	**Controls (n = 38)**
**Patient characteristics**			
Age (years)		28 (23–39)	32 (21–40)
Gender (%)			
	Male	43 (81)	32 (84)
	Female	10 (19)	6 (16)
**Clinical details**			
Leprosy classification (%)			
	LL (lepromatous)	35 (66)	18 (47)
	BL (borderline lepromatous)	18 (34)	20 (53)
Time since diagnosis of leprosy (months)		26 (17–42)	17 (7–34)
MDT treatment duration (months)		20 (13–24)	16 (4–24)
Prevalence of leprosy reactions (%)			
	Type 2 reaction (ENL)	53 (100)	0 (0)
	Type 1 reaction	4 (8)	4 (11)
	Neuritis	11 (21)	16 (42)

Data are n (%) or median (IQR).

The median time since the first episode of ENL in cases was 24 months (IQR 14–36). Seventy-seven percent of cases (n = 41) had chronic ENL and 23% (n = 12) experienced multiple acute episodes. Cases had 2 hospital admissions (IQR 1–3) each lasting an average 35 days (IQR 14–130) whilst controls had 1 admission (IQR 1–1).

### Household income and socioeconomic status

Monthly household income was Rs 6000 (IQR 3600–8100) and 6000 (IQR 3895–8370) for cases and controls respectively. Household size ranged from 2 to 18 persons (median 5 persons, IQR 4–7) and was similar for cases and controls. Daily manual labour was the major source of income and generated a median Rs 5100 (IQR 3525–7925) per month. Small-scale agriculture was a source of income in 37 households, generating a median Rs 750 (IQR 540–1600) per month. Three households received a regular cash income from a family member not living at home, and three received government support (disability allowance or pension). Most patients (92%) lived in a small village. There was no correlation between household assets and household income.

### Treatment-seeking and direct costs

**Figure 1:**
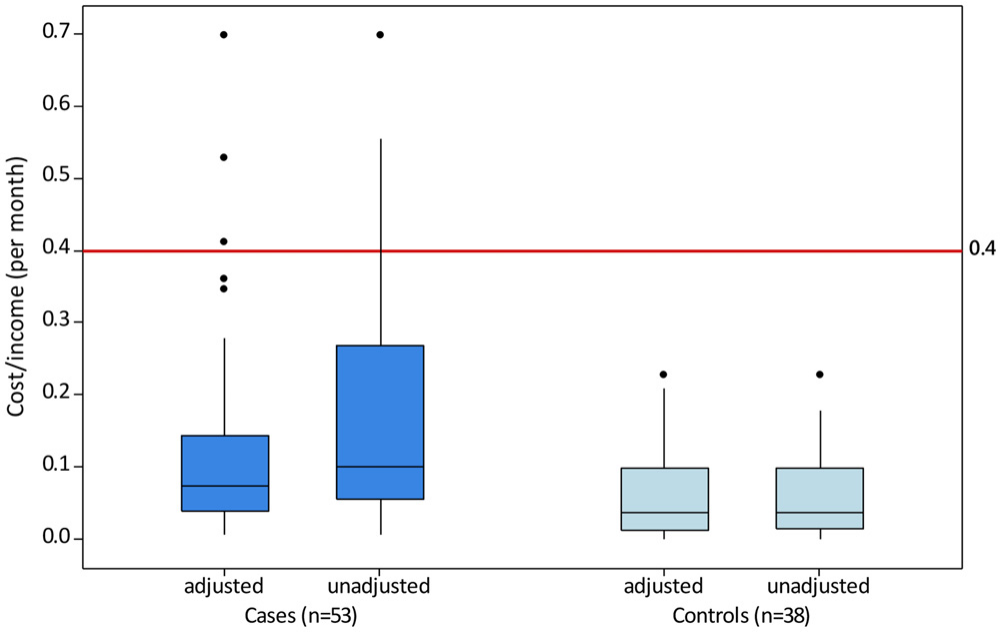
Total direct cost as a fraction of household income (per month) in cases and controls. Whiskers contain 100% of the data, not including extreme values (those data observations which lie more than 1.5*IQR lower than the first quartile or 1.5*IQR higher than the third quartile). The reference line at cost/income = 0.4 indicates the threshold for catastrophic health expenditure.

The total direct cost per month (adjusted) was Rs 465 (IQR 217–738) for cases and Rs 175 (IQR 92–386) for controls, or 7.5% and 3.7% of monthly household income for cases and controls respectively. The monthly total direct cost, as a percentage of household income, was 3.2% higher in cases than controls (Mann-Whitney test; 95% CI 1.2–5.8, W = 2814.5, p = 0.0025). The proportion of households facing catastrophic health expenditure was 11% (n = 6) for cases and 0% (n = 0) for controls. The unadjusted direct cost was Rs 587 (IQR 235–1651) for cases and Rs 204 (IQR 104–248) for controls, or 10.1% and 3.7% of monthly household income for cases and controls respectively. TLM Purulia incurred an average cost per patient of Rs 5026 (IQR 180–27079) for cases (n = 31) and Rs 200 (IQR 45–965) for controls (n = 17) by providing subsidised service to patients (see Fig. 1). This illustrates the extent to which families were unable to cope with the increased treatment costs associated with ENL.

Treatment-seeking in the private sector caused 96% and 99% of total direct costs for cases and controls respectively.

Outpatient services at private health providers other than TLM Purulia were sought by 64% of cases (n = 34) and 47% of controls (n = 18). Traditional healers were consulted by 43% of cases (n = 23) and 29% of controls (n = 11). The median distance from household to traditional healer was 10km (IQR 2–30), compared with 45km (IQR 25–78) for all other private health providers at which outpatient services were consumed. In 41% of patients visiting a traditional healer (n = 14) transport was by foot or bicycle.

Medical and non-medical costs for inpatient and outpatient episodes at TLM Purulia are shown below in table 2. Invoices containing these data were not available for episodes with other healthcare providers.

**Table 2:** Total medical and non-medical costs (per patient) for inpatient and outpatient care at TLM Purulia.

		**Cases**				**Controls**			
		Median	IQR	Mean	sd	Median	IQR	Mean	sd
Distance from home (km)		60	(40–90)	71	(9)	40	(26–49)	42	(5)
**Inpatient care**		**n = 48 (91%)**				**n = 20 (53%)**			
Number of episodes		2	(1–3)	2.4	(1.8)	1	(1–1)	1.1	(0.1)
Medical costs									
	Medicines	297	(164–11542)	5501	(8806)	162	(92–176)	192	(203)
	Other treatment	0	(0–250)	151	(265)	0	(0–250)	106	(168)
	Investigations	200	(0–3585)	1555	(2170)	0	(0–0)	21	(48)
	Admission fees	3363	(1100–16809)	9483	(12075)	1290	(915–1804)	1414	(526)
	**Total medical**	**3712**	**(1703–31373)**	**16690**	**(22558)**	**1525**	**(1125–2186)**	**1734**	**(679)**
	**Total medical (adjusted)**	**2851**	**(1153–7147)**	**6131**	**(8650)**	**1413**	**(993–1585)**	**1398**	**(626)**
Non-medical costs									
	Transport	240	(161–480)	385	(449)	100	(45–140)	111	(94)
	Other	0	(0–0)	6	(43)	0	(0–0)	0	(0)
	**Total non-medical**	**260**	**(161–480)**	**499**	**(863)**	**100**	**(45–140)**	**111**	**(94)**
Total direct cost		4382	(1916–31743)	17081	(22738)	1734	(1348–2299)	1885	(683)
Total direct cost (adjusted)		3158	(1580–7837)	6523	(8760)	1462	(1091–1885)	1550	(581)
**Outpatient care**		**n = 53 (100%)**				**n = 38 (100%)**			
Number of episodes		15	(6–22)	15.7	(10.7)	10	(4–13)	9.7	(7.6)
Medical costs									
	Medicines	663	(295–1386)	839	(679)	139	(49–396)	273	(343)
	Other treatment	0	(0–0)	40	(120)	0	(0–0)	50	(128)
	Investigations	390	(240–485)	377	(185)	185	(130–270)	235	(206)
	Consultation fees	180	(90–230)	200	(159)	90	(60–150)	128	(132)
	**Total medical**	**1210**	**(620–1916)**	**1454**	**(981)**	**493**	**(276–750)**	**687**	**(598)**
	**Total medical (adjusted)**	**1210**	**(620–1916)**	**1397**	**(924)**	**493**	**(276–750)**	**658**	**(559)**
Non-medical costs									
	Transport	1850	(720–3225)	2485	(2653)	780	(323–1625)	1064	(918)
	Other	0	(0–0)	142	(1030)	0	(0–0)	0	(0)
	**Total non-medical**	**1850**	**(720–3330)**	**2627**	**(2879)**	**780**	**(323–1625)**	**1064**	**(918)**
Total direct cost		3002	(1404–5690)	4081	(3578)	1302	(650–2554)	1751	(1406)
Total direct cost (adjusted)		3002	(1404–5592)	4024	(3557)	1302	(650–2406)	1723	(1383)

### Indirect costs

The market economy losses resulting from reduced household productivity due to ENL were significant. The median reduction in monthly household income was Rs 969 (IQR 227–1873) in cases and Rs 60 (IQR 9–186) in controls, or 18.2% (IQR 5.3–33.7) and 1.4% (IQR 0.1–2.6) of monthly household income in cases and controls respectively. On average the reduction in monthly household income was greater in cases than controls by 13.3% (Mann-Whitney test; 95% CI 6.0–21.7, W = 3175, p<0.0001).

The causes of reduced household productivity included physical illness (unwell at home), hospital admission and attendance at outpatient appointments. Physical illness resulted in a loss of productivity in 94% of cases (n = 50) and accounted for a median 55% (IQR 27–80) of the total indirect cost incurred by these households. Hospital admission and attendance at outpatient appointments caused 33% (IQR 12–51) and 15% (IQR 5–60) of total indirect costs respectively. Seventy-six percent of cases (n = 38) received informal care from an average of 1.2 household members. The time diverted by providing care for cases caused a median loss of income of Rs 725 (IQR 233–1500) per household over the duration of ill-health. These data show that ENL affects the productivity of patients and other household members and has a significant impact on household earning potential.

### Total household costs

**Figure 2:**
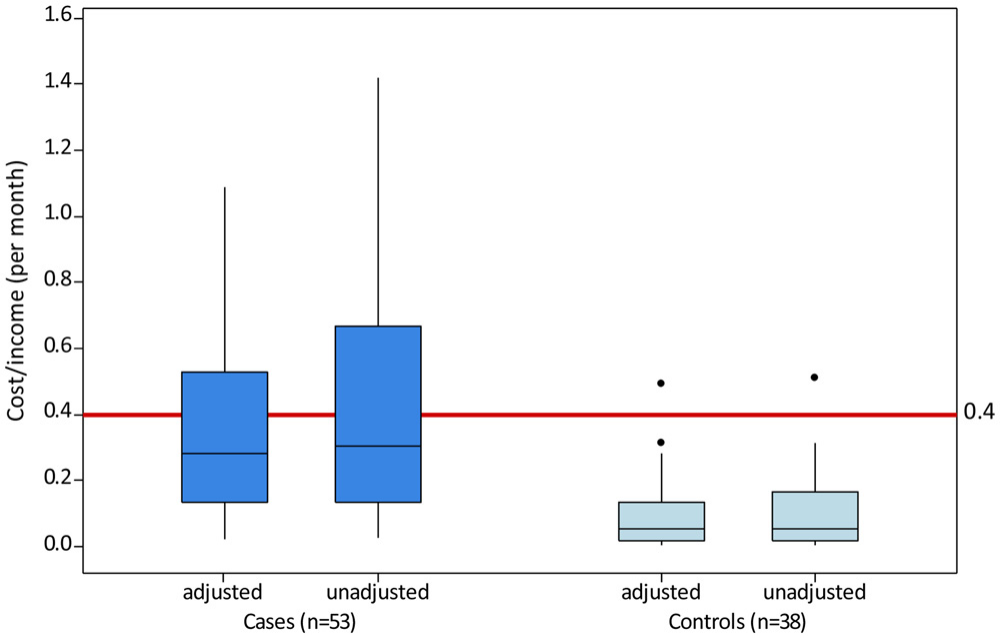
Total household cost as a fraction of household income (per month) in cases and controls. The proportion of households with total costs in excess of 40% monthly household income was 37.7% (n = 20) for cases and 2.6% (n = 1) for controls. Two households (cases) incurred total costs per month in excess of household income (cost/income >1).

Having a family member with ENL impacted on household consumption opportunities. The total household cost per month was Rs 1543 (IQR 681–3175) for cases and Rs 237 (IQR 124–656) for controls, or 27.9% (IQR 13.2–52.6) of monthly household income for cases and 4.9% (IQR 1.7–13.4) for controls. On average the total household cost per month, as a percentage of household income, was 20.8% higher in cases compared with controls (Mann-Whitney test; 95% CI 12.6–29.4, W = 3143, p<0.0001). Fig. 2 displays the total household cost for cases and controls.

The proportion of households with total household costs in excess of 40% monthly household income was 37.7% (n = 20) for cases and 2.6% (n = 1) for controls. Two cases incurred total household costs per month in excess of household income (cost/income >1).

Total household costs consisted of 65% indirect costs and 35% direct costs in cases compared with 21% indirect costs and 69% direct costs in controls (see table 3).

**Table 3:** Summary of household costs for cases and controls.

		**Cases**				**Controls**			
		Median	(IQR)	Mean	(sd)	Median	(IQR)	Mean	(sd)
**Direct cost**									
Inpatient care		n = 49 (92%)				n = 20 (53%)			
	Total inpatient cost	347	(115–1178)	713	(814)	218	(91–650)	409	(456)
Outpatient care		n = 53 (100%)				n = 38 (100%)			
	Total outpatient cost	217	(129–351)	334	(364)	108	(60–313)	277	(436)
Total direct cost		587	(235–1651)	1041	(1060)	204	(104–428)	332	(352)
Total direct cost (adjusted)		465	(217–738)	698	(884)	175	(92–386)	314	(345)
**Indirect cost**									
Total indirect cost		969	(227–1873)	1410	(1419)	60	(9–186)	162	(299)
**Total household cost**									
Total household cost		1882	(681–3826)	2450	(2035)	258	(125–695)	495	(596)
Total household cost (adjusted)		1543	(681–3175)	2107	(1743)	237	(124–656)	477	(585)

Costs are per month per patient (Rs)

### Coping strategies

All patients used cash savings on one or more occasions to pay for treatment. Other coping strategies included selling assets, borrowing money and being gifted money; these were used in 69.8% of cases (n = 37) and 55.3% of controls (n = 21).

Selling assets to generate cash was a strategy used by 32% of cases (n = 17) and 16% of controls (n = 6). Cases sold assets to a median value of Rs 6000 (IQR 2850–17000) compared with Rs 1850 (IQR 825–3125) for controls. Forty-two percent of cases (n = 22) took a loan of median value Rs 5000 (IQR 2000–10250) and incurred costs (interest on the loan) ranging from Rs 0 to 11700. Thirty-two percent of controls (n = 12) took a loan of median value Rs 1000 (IQR 850–4250) and incurred costs ranging from Rs 0 to 18000.

## Discussion

This is the first study to assess the economic consequences of leprosy at the household level.

We show that having a family member with ENL places considerable financial strain on households in rural India. The impact of ENL on households was predominantly through indirect costs, which accounted for 65% of total household costs. By comparison, indirect costs accounted for only 21% of total household costs for controls. This finding highlights the extent to which ENL disrupts the normal functioning and productivity of households, and is consistent with the findings of economic evaluations for other diseases including malaria (indirect costs accounted for 72–94% of total household costs)[8–10] and visceral leishmaniasis (53% of total costs).[11]

Direct costs resulted almost entirely from treatment-seeking in the private sector. Cases incurred greater direct costs, predominantly due to the increased number and duration of hospital admissions required for treatment of ENL. Most cases were unable to pay in full for the services consumed at TLM Purulia which placed the hospital under considerable financial strain.

Continued presentation to specialist (private sector) service providers following integration of leprosy services has been observed in other countries including Nepal and SriLanka.[15,16] This study did not explore the reasons affecting choice of healthcare provider, however access is likely to be an important factor. Traditional healers were consulted by cases and controls more than any other healthcare provider (not including TLM Purulia) and were the closest to the household.

### Limitations

Capturing an appropriate time dimension for the illness was difficult. Our aim was to assess the economic impact of ENL, as a cause of chronic ill-health, on households over many years. We used 3 years as the maximum time dimension for which cost data were collected, acknowledging the risk of recall bias. The accuracy of estimates was aided by the availability of detailed clinical information and cost data from the hospital records at TLM Purulia.

TLM Purulia is a specialist service provider, therefore referral bias may have produced an atypical study population. The high prevalence of ENL overall (58%) and particularly among those with BL leprosy (47%) is unlikely to be representative of the target population. It is likely that patients with severe ENL were overrepresented in this population and the estimates of the economic burden presented in this study are also therefore not representative. However it is possible that patients recruited from this hospital had a higher than average household income (transport costs may prevent poorer patients from gaining access to the hospital) thus lessening the perceived economic impact of ENL.

### Recommendations for further work

Further work is needed to evaluate the economic consequences of leprosy and to validate these findings on a larger scale. Future studies could eliminate selection bias by recruiting patients from the community. Estimates of household costs could be improved if cost data were collected prospectively, although data collection would take a long time and the process may be less efficient with more drop-outs and missing data. In future studies it would be useful to collect data on other comorbidities and their associated costs and in particular to identify any health conditions that might complicate the treatment of leprosy/ENL.

### Concluding remarks

Catastrophic health expenditure is common in low- and middle-income countries, [17] and the high rate of catastrophic spending observed in households affected by ENL in this setting was not surprising. Leprosy is associated with a high level of poverty, and patients with ENL face the double insult of high levels of (predominantly private sector) healthcare use and poor access to financial risk protection (prepayment) mechanisms. Increasing the availability of prepayment mechanisms is needed to reduce the level of out-of-pocket expenditure and protect households from catastrophic payments.[18,19]

Indirect costs account for the majority of total household costs for ENL. Many households in rural India rely heavily on manual labour (agriculture) to provide cash income on a day to day basis and these households are vulnerable to significant reductions in income resulting from ill-health of household members. Thus strategies that aim to develop more robust and sustainable sources of income and protect against income loss would be an important component of reducing household costs.

These findings are relevant to many people. Doctors should advise patients to present early to an established leprosy centre and avoid seeking costly treatments with other private providers. Local providers of alternative and complementary therapies should be engaged and encouraged to refer patients for specialist treatment promptly. Other healthcare professionals including social workers should be aware of the economic burden of ENL so they can fully understand the problems faced by patients and provide appropriate support. These findings are important to academics across a range of disciplines from health economics to clinical medicine. This study paves the way for research to better understand how leprosy impacts on the consumption choices made by families over time. Future cost-effectiveness analyses of interventions for leprosy and ENL that make use of these data on household costs will usefully contribute to discussions on resource allocation. Funding bodies should be made aware of the burden of ENL and the importance of conducting research to better understand this neglected complication of leprosy.[20] Charities and donors need to understand the magnitude of economic burden so that leprosy programmes can be adequately funded. Specifically if control strategies and interventions for leprosy are to be socially viable, it is essential that policy makers acknowledge the significant contributions to household costs of private sector provision and lost productivity. Since household costs of ENL are very high, interventions to prevent or reduce the duration of ENL could be very cost-effective.

## Supporting Information

S1 STROBE Checklist (DOC)
